# The Croonian lecture 2006 Structure of the living cell

**DOI:** 10.1098/rstb.2006.1960

**Published:** 2007-01-24

**Authors:** Iain D Campbell

**Affiliations:** Department of Biochemistry, University of OxfordSouth Parks Road, Oxford OX1 3QU, UK

**Keywords:** structure, macromolecules, cells, microscopy, cell migration

## Abstract

The smallest viable unit of life is a single cell. To understand life, we need to visualize the structure of the cell as well as all cellular components and their complexes. This is a formidable task that requires sophisticated tools. These have developed from the rudimentary early microscopes of 350 years ago to a toolbox that includes electron microscopes, synchrotrons, high magnetic fields and vast computing power. This lecture briefly reviews the development of biophysical tools and illustrates how they begin to unravel the ‘molecular logic of the living state’.

## 1. What is structural biology?

Cells vary widely in size but are, typically, around one-hundredth of a millimetre (10 μm) in diameter. Each cell is surrounded by a thin membrane with a complex interior, packed with many millions of molecules. Although the life we see around us appears very diverse (from ants to elephants! See figure 1), it is remarkably similar at the level of molecules; the exploration of life in the range 10 μm–0.1 nm is thus especially rewarding (the size of an atom is approx. 0.1 nm=10^−10^ m). Such small things cannot be observed with the naked eye, so magnification tools are required to visualize them.

Studies of cell structure, structural cell biology, began when early microscopes, developed around 350 years ago, gave the first glimpse of single cells. Since then, increasingly sophisticated instruments have revealed ever more information about cell structure (Schliwa 2002; figure 2). Visualization of the large molecules (macromolecules) found in cells, structural molecular biology, only began around 60 years ago when atomic resolution models of DNA and proteins were first obtained (protein dimensions are usually more than 1000 times smaller than cells). Like studies of cells, studies of macromolecules have advanced remarkably (figure 2) and high-resolution structures of many thousands of them are now known (Campbell 2002). The protein databank (PDB) is a depository for all the solved structures and a quick browse of the web site gives a sense of their range and beauty, see http://www.rcsb.org/pdb/Welcome.do. Although structural cell biology and structural molecular biology largely evolved as separate disciplines, there is an increasing awareness that they are more and more convergent (Sali *et al*. 2003; Harrison 2004). Ever better resolution can be achieved in studies of intact cells and ever larger structures and complexes can be seen using molecular methods. A new structural biology is emerging that encompasses both approaches.

Advances in all branches of science depend heavily on the development of new techniques^1^ and this is particularly true for structural biology. To illustrate the advances made since the time of William Croone (1633–1684), I will briefly trace the evolution and development of visualization methods. To understand life, we need to know the structure and mechanism of cellular machinery that drives key cellular processes such as cell division, protein synthesis and energy production. To do this, we need to use a range of techniques that give temporal as well as spatial information about the molecules in the crowded environment that exists in and around a cell. The complexity of this problem is clearly illustrated by artistic impressions of the crowded molecular interior of a cell (Goodsell 2005; http://www.scripps.edu/mb/goodsell/illustration/patterson/; Berman *et al*. 2000). This is a static two-dimensional view; in reality, the components are in a constant state of flux, being manufactured, broken down and moving in three dimensions.

As someone trained in physical sciences, much of my emphasis here will be placed on contributions that have passed from physics into the biological sciences, but many other disciplines, including genetics, chemistry and medicine have, of course, also made seminal contributions to our current level of understanding. To indicate what the current level of understanding is, I will expand a little on one area of personal research interest, cell movement on a surface, a fascinating example of the dynamism of the living state.

## 2. The living cell

To set the scene, here are a few quotations from previous eloquent descriptions of the essentials of the living cell:

With the cell, biology discovered its atom …. To characterize life, it was henceforth essential to study the cell and analyse its structure. (Jacob 1973)

a factory that contains an elaborate net-work of interlocking assembly lines, each of which is composed of a set of large protein machines. (Alberts 1998)

a bustling community of macromolecules, like a metropolitan city. (Vale 2003)

the real units of function and structure in an organism are cells and not genes? (Brenner 2003)

to build the most basic yeast cell…you would have to miniaturize about the same number of components as are found in a Boeing 777 and fit them in a sphere just 5 μm across; then somehow you would have to persuade that sphere to reproduce. (Bryson 2003)

Early investigators of cells, including the great Louis Pasteur (1822–1895), were ‘vitalists’, who believed that living organisms have a non-physical inner essence which makes them alive. Although some might still argue that life cannot be completely understood by the usual approaches of molecular biology, which involves separation and dissection of the component parts, there is overwhelming evidence to suggest that life is explicable in terms of the laws of physics and chemistry. As Albert Lehninger (1917–1986) pointed out in early editions of his well-known biochemistry text book

living organisms are composed of lifeless molecules … that conform to all the laws of chemistry but interact with each other in accordance with another set of principles—the molecular logic of the living state. (Lehninger 1975)

The latter half of the twentieth century saw remarkable developments in ideas about biology at the molecular level. Some of these are: monomers (nucleotides, amino acids and sugars), which are strung together to make polymers (DNA, proteins and polysaccharides); a gene (DNA) provides the template for RNA (a close-chemical relative of DNA), which, in turn, specifies the linear sequence of amino acids in a protein; this sequence defines the protein three-dimensional structure that consists of helical and sheet-like substructures; proteins form the main structural and functional components of cells; proteins interact with numerous molecular partners.

Much of the remarkable success of modern cellular and molecular biology methods is due to ‘empirical reductionism’ where, for practical reasons, components of biological systems are isolated, purified, dissected and manipulated to extract information about their structure and properties. It is always important, however, to think about how these isolated components work in the context of the intact living cell. One alternative to the reductionist approach would be to assemble some form of primitive ‘artificial life’ in a test tube. Intensive efforts to do this are underway, but the construction of a minimum life form, apparently requiring around 300 genes, still seems a very long way off (Deamer 2005), not least owing to the difficulty in making catalysts that reproduce themselves. There is also the view that

the spatial organization in a cell is not (entirely) written in the genetic blueprint; it emerges … from the interplay of genetically specified molecules … constrained by heritable structures. (Harold 2005)

This idea, that we need more than genes to make life, is consistent with the ‘law’ proposed by Rudolf Virchow (1821–1902) ‘Omnis cellula e cellula’ (every cell comes from a pre-existing cell), but this might make the task of reproducing life harder than anticipated!

Life is dominated by natural selection. Evolutionary forces are clearly apparent in cellular structures. Biological systems utilize components that have been found, by chance, to be effective and robust. The ribosome, the machine that synthesizes proteins using an RNA template, has been retained in very similar form by all kinds of life. Mitochondria, the energy-providing compartments in many cells, are thought to have arisen from what were once separate free-living organisms. Structural molecular biology has also shown that a limited number of protein structural folds or domains are used repeatedly in various ways. Certain structures were apparently found to make effective and versatile scaffolds and then these were retained and exchanged between organisms (this will be discussed further under ‘cell migration’; figures 3 and 4).

This lecture emphasizes biophysical studies at the level of the single cell. It is worth pointing out, however, that higher levels of organization, as observed in multi-cellular organisms like animals, emerge from the blueprint provided by a single cell. Familiar characteristics exhibited by animals, including structure, response to stimuli, and reproduction, are all programmed by a single cell. Even sophisticated higher level properties, such as consciousness, arise from the ways in which our neuronal cells are connected together.

## 3. The evolution of structural cell biology

Man has long been interested in the structure (anatomy) of living organisms. Early pioneers, largely based in Padua, were Andreas Vesalius (1514–1564), who wrote one of the first textbooks on anatomy, Hieronymus Fabricius (1537–1619), who was Galileo's physician, and William Harvey (1578–1657), who discovered the circulation of blood. Dissection of tissue structure to the level of individual cells eluded these pioneers because the available tools were rudimentary. Our understanding of living systems began to improve when a contemporary of Croone^2^, Antonie van Leeuwenhoek (1623–1723), used a single lens microscope that he had ground himself to observe pond water and a variety of other liquids; he noted

I … saw, with great wonder, that … there were many very little living animalcules, very prettily a-moving. (Leeuwenhoek 1683; figure 2*a*)

The study of single cells, a word first used by Robert Hooke (1635–1703), thus began. Microscopy improved in the next 200 years and Ernst Abbé (1840–1905) developed a theory showing that the resolving power of microscopes was approximately proportional to half the wavelength of the observing light; for blue light, this corresponds to a resolution of around 200 nm. Abbé's collaborators, notably Carl Zeiss (1816–1888) and Otto Schott (1851–1935), improved lenses to the point where they could achieve the calculated resolution limit. Pioneers also improved selective staining procedures that allowed them better to visualize different cellular components and, by the end of the nineteenth century, many important features of cell structure and neuronal networks were beginning to emerge (Cajal 1906).

Improvements continued to be made in optical microscopes by enhancing the contrast between different cellular substructures (Zernike 1953), but in the early twentieth century the ability to view cells remained limited. Albert Claude (1899–1983) summarized the situation in his Nobel lecture of 1974:

Until 1930 or thereabout biologists … were permitted to see the objects of their interest, but not to touch them; the cell was as distant from us, as the stars and galaxies were from (astronomers). More frustrating was that we knew that the instrument at our disposal, the microscope—so efficient in the 19^th^ century–had ceased to be of any use, having reached, irremediably, the theoretical limits of its resolving power. (Claude 1975)

This situation was changed by the introduction of the electron microscope. Electrons are particles but act like waves with a wavelength which is inversely proportional to their velocity. Lenses for electrons can be made by bending the electron path with applied magnetic or electric fields. Based on this principle, the first electron microscopes were constructed in the 1930s (Ruska 1986). These have a theoretical resolving power much better than 1 nm but, in practice, this level cannot be readily achieved for biological samples. By developing methods to ‘fix’ cells, with chemicals such as glutaraldehyde, and to stain them, with metals like osmium and uranium, Keith Porter, Albert Claude, George Palade and others were able to discover many cellular substructures and compartments using the electron microscope (Palade 1975).

Like all methods, the electron microscope has limitations. These include severe sample damage caused by the electron beam in evacuated sample chambers and low contrast between different parts of the sample. There have been a number of important recent technical advances in electron microscopy (EM) that have had a significant impact on studies of cell structure (see also molecules discussed below). These techniques include rapid freezing of an aqueous sample, avoidance of metal stains and sophisticated computer programs that can analyse and average noisy images. The background variation, or ‘noise’, arises because very low electron irradiation doses have to be used to avoid destroying the sample. Another advance came from the application of ‘tomography’ methods, which were first used to look at the structure of viruses in the 1960s (Crowther *et al*. 1970). A series of images of an object are taken at different angles to the electron beam. A three-dimensional image can then be reconstructed from these angle-dependent images. By combining these approaches—frozen hydrated samples, low doses, tomography and data analysis—detailed images of the structure of cells have now been obtained (Nickell *et al*. 2006). This technology allows many structural features of a cell, including ribosomes, actin filaments and membrane structures, to be visualized at a resolution of around 2 nm (Baumeister 2004), although most of the other molecules in the cell are still invisible and/or unrecognizable (figure 2*b*).

X-rays, like electrons, can have wavelengths short enough to give less than nanometre resolution, but the construction of X-ray lenses is a formidable problem. Some progress is, however, being made with longer wavelength ‘soft’ X-rays and interesting images of cells are beginning to be produced (Attwood 2006).

Light microscopy has also undergone a remarkable renaissance in the last 20 years. Certain ‘fluorescent’ molecules absorb light at one wavelength and re-emit it at a different, longer wavelength. Beautiful images of fluorescent macromolecules in their cellular location are becoming familiar, as are ‘movies’ of living cells, obtained using time lapse photography (Dunn & Jones 2004). These developments have been enabled by improved fluorescent molecules (Zhang *et al*. 2002) and new microscope technology. One example of a fluorescent molecule that is having a major impact is green fluorescent protein (GFP) (Tsien 2003). This occurs naturally in some jellyfish and the fluorescent centre is produced by the protein itself; GFP can be attached specifically to molecules inside cells and visualized in living cells.

An example of an important technological improvement in light microscopes was the development of confocal methods. The idea of reducing unwanted light scattering from the specimen by scanning a small spot of light and observing the fluorescent emission was demonstrated in the 1950s by Marvin Minsky, but was not developed commercially for many years (Amos & White 2003). Further developments include total internal reflection fluorescence microscopy, where only a thin layer of a specimen near a surface is observed; a light beam reflected from a surface induces a thin layer of light (approx. 100 nm thick) on the opposite side of the reflecting surface and this can be used to excite fluorescent molecules selectively (Schneckenburger 2005). In a modification of the confocal microscope, two light beams can be made to interfere with each other, so that they generate exceptionally small scanning spots (Garini *et al*. 2005); this approach has taken the resolution of microscopes to better than 100 nm, well beyond the theoretical ‘limit’ calculated by Abbé.

With each technical development, our view of cell structure has been extended (Schliwa 2002). We now have a bewildering amount of information about the complexity and structure of cells and their contents. It should be recognized that much of the technology that we use to observe cells—fixation, staining and even GFP—perturbs what is observed. This is, of course, a well-known philosophical as well as practical problem in science, but there is much reason to be optimistic: the history of cell-structure studies clearly illustrates how science advances in cycles of technical development, increased knowledge and improved models.

## 4. The evolution of structural molecular biology

Biological macromolecules, such as proteins and nucleic acids, are constructed from atoms and bonds with dimensions of around 0.1 nm. To understand how macromolecules operate, we need to know how the atoms come together to form their precise and intricate shapes, thus forming structural scaffolds, catalysts and a variety of elaborate machines. Although EM has the capacity to give at least 0.1 nm resolution, it is severely limited by practical difficulties and alternative techniques were, fortunately, also developed. There are now three main experimental methods that can give atomic resolution structural information—X-ray crystallography, electron crystallography and nuclear magnetic resonance (NMR).

The serendipitous discovery of X-rays by Wilhelm Röngten (1845–1923) in 1895, followed by the structure determination of crystalline common salt in 1913 by Lawrence Bragg (1890–1971), led to the discipline of X-ray crystallography. Small crystals of macromolecules are irradiated with a beam of X-rays (wavelength around 0.1 nm) to produce a diffraction pattern, which is then interpreted in terms of a three-dimensional atomic model. This method has been spectacularly successful in recent years, although the first steps, by the pioneers like Max Perutz (1914–2002) and John Kendrew (1917–1997) were painstakingly slow (Rossmann 1994). A major early stumbling block was to recover essential phase information about the waves making the diffraction pattern (figure 2*c*). Several ways of solving this ‘phase problem’ have now been devised, including one where naturally occurring sulphur in a protein is substituted by another element, selenium. X-ray crystallography and its lesser used cousin, neutron crystallography, have now been used to solve more than 33 000 macromolecular structures: see http://www.rcsb.org/pdb/Welcome.do. Protein structures used to take decades to solve, but now new structures can often be derived in a few days; the hard part is usually to obtain crystals of pure protein.

More recently, two other techniques have been shown to be capable of giving atomic resolution information about biological macromolecules. One of these is electron crystallography, where electron microscope images of thin two-dimensional crystals, collected at different angles to the electron beam (tomography), are combined with electron diffraction information to give a three-dimensional structure. This approach was used to give the first model of the structure of a protein from a salt-loving bacterium (Henderson & Unwin 1975); it was shown to have seven membrane-spanning helices. The number of structures obtained by this method is still relatively small, but there are some important examples of its use, especially with membrane proteins (Gonen *et al*. 2005).

The other method that can give atomic resolution structural information is NMR, a technique first demonstrated independently in 1946 by Felix Bloch (1905–1983) and Ed Purcell (1912–1997). This method observes nuclei that have magnetic dipoles. These ‘resonate’ in the radiofrequency range (10–1000 MHz) when they are placed in a strong magnetic field. The resonance frequency is exquisitely sensitive to the electrons around the nuclear dipole, so that individual resonances can be resolved and assigned to particular atoms in a macromolecule. Proteins are made from strings of amino acids. There are only 20 amino acids so that even a small protein will contain several amino acids of one type in its sequence. An NMR spectrum of a small protein will usually be able to resolve all the different nuclear resonances from different groups and to assign them to specific positions in the amino acid sequence. Assignment is done by identifying the amino acids' neighbours in the known protein sequence (just as the three ‘e's in the word ‘sequence’ are made unique when neighbouring letters are considered). Thousands of specific short-range interactions (approx. 0.3 nm) between nuclear dipoles can be detected in a protein. This information, combined with ‘molecular dynamics simulations’ (see next paragraph), allows the structure of macromolecules to be calculated^3^ (Wüthrich 2003). An advantage of the method, which has now solved over 5600 structures, is that the protein is simply dissolved in water; crystals are not required and there is no ‘phase problem’. There is, however, a practical size limit because the resonances can no longer be resolved and assigned when the macromolecules become very large. The method works best for determining the structure and dynamics of relatively small macromolecules of about 200 amino acids in length (approx. 22 kDa); this limit can, however, be extended to over 50 kDa by extensive and expensive isotope labelling (e.g. substituting ^12^C by ^13^C, ^14^N by ^15^N and many ^1^H by ^2^H; Kay 2005).

The above three methods, crystallography, EM and NMR, depend heavily on computational methods to derive, refine and visualize models of the macromolecule or complex being studied. Computer modelling is also very useful for giving insight into the structure of proteins with a close relative of the known structure. Modelling can also be used to explore ways in which two molecules might dock together, an approach much used by the pharmaceutical industry in their quest for new drugs. Simulations of molecular motions in a computer, by allowing atoms to move under the influence of various energetic restraints, can give considerable insight into possible macromolecular structures and how the various parts can move with respect to each other. Such simulations are usually carried out by considering a ‘box’ containing the molecule(s) of interest plus solvent water. The possible trajectories of the atoms in the box are calculated, one very short step (10^−15^ s) at a time, using laws of physics that were first described by Isaac Newton (1643–1727). An example of this approach is the exploration of how proteins can fold from an extended chain to a well-defined structure (Vendruscolo & Dobson 2005). These ‘molecular dynamics’ simulations are also very important in refining structures derived from experimental information. A difficulty is that the time-scales that can be simulated, even using very powerful computers, are relatively short (approx. 10 ns) compared with the time-scales of most molecular interactions in the cell. This limitation has been relaxed to some extent recently by the realization that larger time-step sizes can often be used if suitable approximations are made (Bahar & Rader 2005).

There have been exceptional technical developments and improvements in structure-determination methods over the last 30 years. The improvements in computers are familiar to us all, but X-ray sources, NMR spectrometers, microscopes and data collection procedures are all orders of magnitude better than they were; for example, the signal-to-noise ratio obtained in a single scan NMR spectrum is now several orders of magnitude better than it was 40 years ago—a result of higher field strengths, better electronics and better data collection methods. We also must not forget that molecular biology methods have improved dramatically; almost any specified protein can now be produced by expressing its gene in host cells, often in simple bacteria, and the sequences of most important proteins are now known owing to genome-sequencing projects.

These various improvements have led to an extraordinary growth in structural information in the last 25 years. Representative examples of most classes of existing protein structures are already known. Even systems thought to be particularly intractable, such as proteins embedded in membranes, are yielding to sustained efforts (see http://blanco.biomol.uci.edu/membrane_proteins_xtal.html). The key structural features of several of the essential, multi-component molecular machines in cells have also been solved. Examples include muscle action (Geeves & Holmes 2005), the transcription of DNA into RNA (Armache *et al*. 2005) and the synthesis of proteins from an RNA template on the ribosome (Moore & Steitz 2005; figure 2*d*). In many cases, the models for these complex machines have been obtained using combinations of all the molecular structural tools mentioned above—crystallography, EM, NMR and molecular modelling. This astonishing success certainly does not mean that structural molecular biology is reaching an end. While structures of complexes like the ribosome, which are relatively stable and resistant to the rigours of isolation and purification, have now been solved, it is clear that many of the networks of protein–protein interactions which make essential complexes are transient. It will, therefore, be much harder to build up a picture of how these dynamic systems operate.

## 5. Bridging the gaps

I have outlined current ways of visualizing cells (μm) and molecules (0.1 nm; figure 2). How can we fit all this together to obtain an operational model of the living cell? The methods discussed so far still leave many gaps in our knowledge about the ‘bustling community of molecules’ in the cellular environment. Filling these gaps remains a major technical challenge.

EM of cell structures has already been mentioned and is a tool that can give structural information over a wide range (nanometre to micrometre). Large macromolecular complexes can be studied by EM using ‘single particle’ analysis; the particles are embedded in ice formed by rapid freezing and images are taken using a weak dose of electrons. Many thousands of noisy images are collected, analysed and averaged to obtain a three-dimensional reconstruction of the object. This method still cannot give us atomic resolution images, but resolution better than 1 nm is possible (Frank 2006). This method is especially powerful when used in combination with other techniques; for example, high-resolution X-ray structures of components can be fitted into low-resolution EM images of multi-component complexes. This is the one way of finding out about the operation of an assembled cellular machine like a muscle (Geeves & Holmes 2005).

Other techniques that span structural studies of cells and molecules include those that can observe the location and properties of a single molecule at a time. Early success was achieved using ‘patch clamp’ methods, where ions passing though a single membrane-spanning channel were detected electrically (Sakmann 1992). Atomic force microscopy (Binnig & Rohrer 1983), where a sharp probe is scanned in a controlled way over molecules on a surface thus tracing their shape, has become a valuable and flexible tool both for looking at large molecules and studying various interactions (Horber & Miles 2003). Scanning EM can also be powerful in the 100 nm range (Wanner *et al*. 2005). Another way of manipulating single molecules is to use the small, but measurable force that light exerts on an object. In 1970, Arthur Ashkin noticed that he could use laser light to trap small latex beads (Ashkin 1997). This technology, often referred to as ‘optical tweezers’, can manipulate single molecules and measure the forces they exert.

The innumerable interacting networks among cellular components in a cell are sometimes called the ‘interactome’ (Cusick *et al*. 2005). These interactions are the key to what makes cells ‘alive’. What experimental tools can be used to define them? Cell fractionation and analysis of the contents of the fractions are a first step; an early example was enzyme assays of fractions obtained after ultracentrifugation (Duve 1975). The fractionation approach is now generally called ‘proteomics’ when applied to studies of protein components of the cell. Protein mixtures can be resolved by spreading them out in two dimensions by electrophoresis; separation in one dimension is based on charge, the other on size. Once resolved, the different proteins can be identified by measuring their mass accurately in a mass spectrometer. A recent review classifies the location of thousands of different proteins in different cellular compartments using such methods (Yates *et al*. 2005). Microscopy can also determine the location of particular molecules if they are tagged with visible labels and there are ambitious projects to tag all the proteins in a genome with GFP (Simpson & Pepperkok 2003).

While a catalogue of components, with their cellular locations, is essential, we also need to know about the dynamic organization of the ‘elaborate network of interlocking assembly lines’. One way forward is to map the various interactions using ‘screening’ methods where a large number of potential targets can be investigated rapidly. One such is the ‘yeast two-hybrid’ screen where an interaction between proteins is detected by switching on a gene that results in a colour reaction. This approach has been used to identify over 4000 protein–protein interactions in the nematode worm, *Caenorhabditis elegans* (Li *et al*. 2004). Other promising methods for identifying multiple interactions include protein ‘microarrays’ (Bertone & Snyder 2005) and large-scale affinity purification (Gavin *et al*. 2006).

Once potential interacting partners have been identified in screens, we need to quantify the interactions using a variety of biophysical tools (Harding & O'Shea 2003; Piehler 2005). Among these is a sensor device (surface plasmon resonance) that monitors changes in mass at a surface when two components interact (Karlsson 2004). Measurements of interactions can also be carried out at the single molecule level using fluorescence microscopy (Kelley *et al*. 2001). For example, if two suitable fluorescent probes come within approximately 10 nm of each other, the fluorescence emitted by one is perturbed by the presence of the other. This ‘fluorescence resonance energy transfer’ can be used to detect interactions between molecules and measure their separation in a living cell (Michalet *et al*. 2003). Another way to define macromolecular interactions is NMR (Clarkson & Campbell 2003). The advantage here is that when two molecules are added together in solution, induced perturbations in the NMR spectra can be identified and mapped onto the three-dimensional structure to show exactly where the interactions take place.

My emphasis, so far, has been on physical methods, but it has also been suggested that

‘mathematics is biology's next microscope—only better!’ (Cohen 2004)

Mathematics can certainly make contributions in a number of ways. One is to give a robust statistical basis for computer-based analyses of the growing databases of sequences, structures and interaction networks. Web-based tools in ‘bioinformatics’ are now a major part of modern cellular and molecular biology (e.g. http://www.ebi.ac.uk/). These allow exploration of the large and rapidly growing biological databases to extract information about sequences, structures and interactions. A recent promising extension is the systematic analysis of protein structures to make predictions about protein–protein interactions (Aloy & Russell 2005). Other contributions of mathematics already mentioned include image analysis, molecular simulations and molecular modelling.

A theme of this lecture is the drive towards visualization of the workings of a living cell. We live in an age where the genome sequences and structural properties of most of the component parts are known, yet the complexity of dynamic cellular networks is bewildering. It is therefore timely to try to employ mathematical methods to help, using an approach often now called ‘systems biology’ (Aderem 2005). Among the goals would be the explanations for how properties, not apparent in the individual parts, can ‘emerge’ from a complex assembly. Mathematics can help here by using sets of equations to describe a network of interacting components. The predictive and explanatory power of such models can, however, only be as good as the quantitative information (about rate constants and concentrations of the interacting components) that they rely on. Close cooperation between modellers and experimentalists should encourage the acquisition of critical quantitative data and enable the validation of models as they are constructed. In a recent example from this laboratory, it was possible to use a combination of equations and experiments to deduce how a single bacterial cell can develop into two different cell types—a spore and a mother cell—in response to stress (Iber *et al*. 2006); this example illustrated how mathematical modelling can guide experiments and give fresh insight into the functioning of whole systems.

## 6. An example—cell migration

To illustrate how the complex molecular interactions in a living cell can be unravelled, I will briefly discuss cell migration. All the molecular and cellular tools mentioned above have been brought to bear on this topic. Cell motility is a recognizable property of a living system and van Leeuwenhoek quickly realized that the tiny moving objects he saw in his microscope were alive. The ability of a cell to move is a necessary feature of many essential processes in life and disease, including the development of an embryo, the repair of tissue and the spread of cancer. Key questions we need to answer are: ‘how do cells move?’ and ‘how do they respond to stimuli?’ Much progress has already been achieved and a large amount of information on this topic is already available in the literature (Bray 2001; Ridley *et al*. 2003; Webb *et al*. 2005) and Websites (http://www.cellmigration.org/index.shtml). I will concentrate here on eukaryotic cells that can crawl on surfaces, such as fibroblasts (skin cells) and neutrophils (a type of white blood cell).

Figure 3 contains a simplified illustration of a cell moving on a surface. Neutrophils migrate from the blood stream to infected tissues to hunt out and destroy unwanted invaders when they ‘smell’ danger. The danger signal may be a peptide derived from bacteria; this is detected by a cell surface protein receptor, which then transmits a signal to the cell interior, resulting in cell movement in a particular direction. The chief forward propulsion force comes from the directed addition of globular actin monomers to a growing actin filament (F-actin). This actin assembly process is driven by hydrolysis of adenosine triphosphate, the main fuel of life. Another key feature of cell propulsion in crawling cells is the formation of contacts, called focal adhesions, between the extracellular molecules, the extracellular matrix and the actin filaments. These provide the ‘grip’ that allows the cell to drag itself forward. The cells extend a thin (approx. 200 nm thick) leaflet of cytoplasm, called a lamellipodium, at the front of the cell, where new focal adhesions are formed. At the same time, focal adhesions at the rear of the cell dissolve and the actin filaments retract. Without an external signal, the cells stop moving and go into a quiescent state. A few highlights and key features of the molecular processes involved in cell migration, particularly focal adhesion formation, follow.

### (a) Structural cell biology

Techniques that visualise cells have been used to obtain striking movies of migrating cells; these show not only the movement of the entire cell, but also the paths of specific proteins within the cell (see http://www.cbrinstitute.org/labs/springer/lab_goodies/springer_teaching_movies.html; http://www.cellmigration.org/science/sci_movies.shtml). The location of particular proteins can be detected using a microscope, provided that they are specifically labelled with GFP or an antibody, a protein derived from the immune system selected to bind to the target protein. This kind of microscopy has detected and classified numerous proteins associated with focal adhesions. Combinations of fluorescence and electron microscopy can be used to measure structural features of a migrating cell; for example, fluorescence can be used to follow the dynamics of filament assembly while EM of metal-coated replicas can give details of the structures formed (Kandere-Grzybowska *et al*. 2005). Mechanical forces, generated while cells migrate, are important for maintaining a healthy cell (Matthews *et al*. 2006) and these forces can be monitored using optical tweezers (Jiang *et al*. 2003). Mathematical modelling has also been a useful tool for predicting aspects of cell migration including protrusion dynamics (Mogilner 2006).

### (b) Structural molecular biology

Structural studies of molecules have made significant contributions to all aspects of cell migration by determining the structure and interactions of the various molecules involved. More than 50 different proteins have been found associated with focal adhesions (Zamir & Geiger 2001) and some key examples are shown in figure 3*e*. These proteins have a wide range of functions. Particularly important are integrins, large adhesion receptors which are formed from *α* and *β* protein subunits that span the plasma membrane. Integrins have been shown to be essential in functions ranging from embryo development to cell death (Hynes 2002; Ginsberg *et al*. 2005). Integrins adhere to the stiff structures, like collagen, in the extracellular matrix, and link them to the relatively rigid, although dynamic, actin filaments inside the cell. The integrin-mediated linkages between collagen and actin are not direct and there are bridging proteins, such as fibronectin, outside the cell and talin, filamin and paxillin inside the cell (figure 3*e*). Remarkably, we now have structural information about most of the molecules associated with focal adhesions (figures 3 and 4). One example where structure determination methods have given great insight is the growth and control of actin filaments; the available structures of numerous protein complexes show how the assembly and disassembly of actin monomers is controlled and how branch points in the filaments are generated (Paavilainen *et al*. 2004; Egile *et al*. 2005). Another elegant example of the power of modern structural molecular biology is given by studies of integrins (figures 3*e* and 5). Structure determinations of various integrins and fragments, using all three of the available high-resolution tools—crystallography (Xiong *et al*. 2002; Xiao *et al*. 2004), EM (Adair *et al*. 2005) and NMR (Vinogradova *et al*. 2002)—have taught us much about how they bind to their substrates and how their affinity is switched between ‘on’ and ‘off’ states by structural changes (Campbell & Ginsberg 2004; Luo & Springer 2006).

### (c) Modular proteins

A general feature of focal adhesions and most signalling complexes is that the constituent proteins are modular—constructed from several modules or domains. Some examples of module structure are shown in figure 4. These modular units are found repeatedly in many different proteins (Campbell 2003). Cells construct all their machinery from only a few thousand different module types. Consider one example, the Fn3 module: there are over 3000 different proteins in the Interpro database (http://www.ebi.ac.uk/interpro/) that contain at least one copy of an Fn3 module. As shown in figure 3, one of these modules binds to integrins. Modules like Fn3 can be recognized at the sequence level, although less than 25% of their sequences may be conserved between different versions. There is good evidence that all sequence-identified Fn3 modules have similar three-dimensional structures. Modular units in proteins are usually relatively rigid structures and most of the observed flexibility in the intact proteins and their complexes arises from movements in the linkages between domains. A rearrangement of domains is often used to produce a change in protein activity; e.g. in figure 3, both Src and integrins (figure 5) undergo extensive domain rearrangements when they go from the ‘off’ to the ‘on’ states (Campbell 2003).

### (d) Flexible protein regions

The idea that proteins form well-defined three-dimensional folds, and that they are modular, has become relatively familiar (figure 4). Another feature that is becoming increasingly apparent is that many segments of proteins are unstructured when they are not associated with another macromolecule (Fink 2005). Careful analysis of protein databases, together with experimental evidence, indicates that a large fraction (more than 30%) of the proteins coded for in various genomes contain disordered regions. These unfolded regions do, however, play an important role in the formation of signalling complexes. The cytoplasmic tails of integrins are a good example: they can form complexes with a wide range of different proteins including talin, focal adhesion kinase and the actin cross-linking protein filamin. Single amino acid substitutions in integrin tails can be lethal and the way that the tails bind to their partner proteins is fine-tuned by adding or removing phosphate groups (Campbell & Ginsberg 2004; Wegener *et al*. in press).

### (e) Communication and signalling

Extracellular signals are detected by protein receptors that span the cell membrane. Actin filaments assemble in a particular direction. Focal adhesions form and dissolve in response to signals received from their environment. Integrins can change their affinity and communicate with the interior of the cell by undergoing structural changes. These are all examples of communication and signalling. Cells have developed a number of ways of sending signals. One common induced change inside the cell is the addition of a phosphate group, by a kinase, to an amino acid in an intracellular protein (Stoker 2005). The added phosphate group changes the types of interactions and reactions that take place. Two kinases are shown in figure 3*e* (Src and FAK); each has a kinase domain (blue pentagon) and two other domains that mediate protein–protein interactions (figure 4). These enzymes are localized at the cell membrane. Phosphate additions by kinases are balanced by their removal by phosphatases and the balance can be tipped one way or the other by slight environmental changes. Calcium ions are another common intracellular signal; their concentration can be increased locally by opening membrane channels. Calpain is an important protein in the regulation of focal adhesion formation. On receiving a calcium signal, it cuts some of the proteins in focal adhesions into pieces, thus causing the focal adhesion to dissolve at the rear of the cell, facilitating forward movement (figure 3*d*; Franco *et al*. 2004). Another set of important signalling proteins is the Rho family, relatively small proteins that can exist in an ‘off’ or an ‘on’ state (Jaffe & Hall 2005), depending on whether a guanine nucleotide di- or triphosphate is bound. Rho proteins are essential for promoting actin polymerization and members of this protein family are found in the ‘on’ state at the front edge of migrating cells. Yet another important family of proteins involved in signalling are ones that act on lipids in the membrane that surrounds the cell; examples are inositol kinase enzymes (e.g. PI3K and PIPK) that locally phosphorylate lipids which then help recruit proteins to specific regions of the cell membrane (figure 3*e*).

### (f) Integrins adjust their affinity by structural changes

A key feature of cell migration and focal adhesion formation is the ability of integrins to exist in different affinity states that can be influenced by signals both from outside and inside a cell. For example, when a neutrophil in the blood stream detects a problem it first slows down by ‘rolling’ on the surface of the blood vessel; it then switches integrins to a high affinity state and goes through the vessel wall to seek out and destroy the problem. There is a body of evidence to suggest that the ‘on’ state which binds tightly to surfaces corresponds to the relatively straight orientation shown in figures 3*e* and 5, with the membrane-spanning regions of each subunit separated. In the low affinity ‘off’ state, however, the extracellular region of the integrin is compact and bent and the two membrane-spanning regions are close together. The nature of this ‘inside-out’ activation process has become much clearer recently and a number of key intracellular players have been identified, including talin, one of the proteins that couples the cytoplasmic tails to F-actin (Campbell & Ginsberg 2004; Luo & Springer 2006; figure 3*e*). In a recent study from this laboratory, we obtained the structure of an integrin tail in complex with talin. We designed and made amino acid changes to produce talin variants that fail to support integrin activation. These results revealed the unique and highly specific nature of the talin–tail complex. The integrin tail can also interact with numerous other proteins, but talin has unique structural properties that bind the tail near the membrane. This promotes the separation of the tails and thus integrin activation (Wegener *et al*. in press).

### (g) Summary

The above brief and incomplete description of some of the complex events going on when a cell migrates is intended to give a glimpse of the current state of knowledge. Cell migration involves numerous proteins with weak, dynamic interactions orchestrated in time and space that result in cell movement in a specified direction. The task of the structural biologist is to explain observations made at the cellular level (micrometer) in term of detailed interactions formed by protein interfaces at the sub-nanometre level. This can now be done in certain cases; e.g. talin interactions with integrin tails lead to an increase in integrin affinity. We are at a stage where most of the key players in particular processes, e.g. focal adhesion formation, are known but the numerous competitive interactions in the cell and the fine-tuning achieved by phosphorylation and protein cleavage are not yet very well understood.

## 7. Conclusion

The history of single cell investigation, begun over 300 years ago, has shown that the more we examine cells and their constituents the more remarkable they seem. Innumerable carefully balanced processes—self-assembly and disassembly, phosphorylation and dephosphorylation, synthesis and breakdown—are continually in play and the balance is adjusted by small changes in environment and energy flow. Our main challenge is to discover how all these components work together in a concerted way.

How can this growing mountain of facts be assimilated, and where will the new ideas come from that will help us gain a broader perspective? (Bray 2003)

Can we define the ‘molecular logic of the living state’? Molecular and structural biology have been extraordinarily successful and it has been argued that

the molecular paradigm, which so successfully guided the discipline throughout most of the 20th century, is no longer a reliable guide—it has run its course. (Woese 2004)

In my view, the ‘mountain of facts’ is not yet complete enough to properly formulate the new integrated vision of the living world that Carl Woese and all of us aspire to. Much progress has been made but we have a continuing need to create better tools, experiments and models before we can fully unravel the complex machinery of life. The current way forward is likely to be a modular approach where we try to understand one set of protein interactions, e.g. focal adhesion formation, and then extrapolate that understanding to the whole system.

When we have established sufficient level of understanding and success at our chosen level, we can reach out to other levels. (Noble 2006)

## Figures and Tables

**Figure 1:**
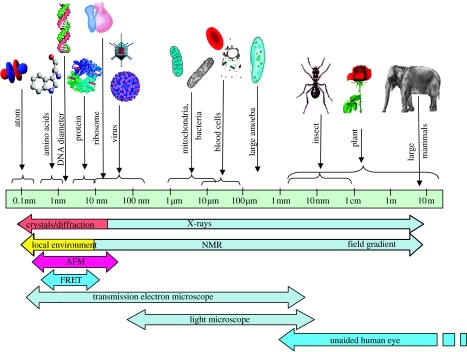
Illustration of the dimensions of some life-related systems on a logarithmic scale (powers of 10). The range visible by some of the different methods mentioned in the text is indicated by horizontal arrows. X-rays and NMR can be used to look at human anatomy in hospitals using tomographic methods, so these are shown as having the capacity to view a very wide range of dimensions. Their application at the nanometre level and below is, however, the only aspect dealt with in the text. FRET refers to ‘fluorescence resonance energy transfer’ and AFM to ‘atomic force microscopy’.

**Figure 2:**
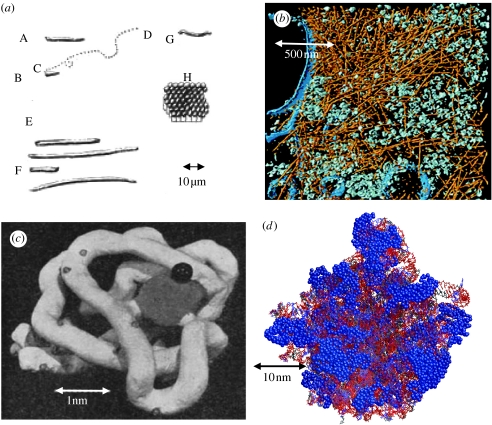
An illustration of advances in structural cell biology (*a* and *b*) and structural molecular biology (*c* and *d*). (*a*) Drawings of ‘animalcules’ seen by van Leeuwenhoek in a single lens microscope (figure 3 in the reference Leeuwenhoek 1683). (*b*) Visualization of the actin network (orange colour) and cytoplasmic complexes (light blue colour) in a *Dictyostelium* cell, embedded in vitrified ice and viewed by electron microscopy (Baumeister 2004). (*c*) A photograph of the first model of a protein, myoglobin, derived from X-ray diffraction data. The polypeptide chains are white and the haem group is shown as a grey disc (Kendrew *et al*. 1958). (*d*) The large subunit of the ribosome (PDB accession code 1ffk) is composed of two strands of RNA (shown as grey and brown wire models) and many different protein chains, shown in blue. Several of the proteins were not seen in this crystallographic structure but can be detected using cryo-electron microscopy. The dimension bars are approximate. Note that the resolution of the myoglobin model, approximately 0.5 nm, was only enough to show the general path of the polypeptide. The much larger ribosome, obtained some 40 years later was obtained at a resolution of around 0.25 nm (Ban *et al*. 2000), sufficient to observe individual amino acids and bases. (*a*–*c*) are reproduced with permission. (*d*) was produced using the computer visualization program Rastop (http://www.geneinfinity.org/rastop/manual/index.html).

**Figure 3:**
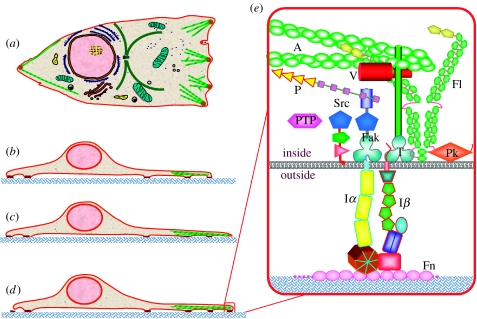
(*a*) A representation of a cell showing some intracellular organelles such as mitochondria (blue-green) and the nucleus (pink); (*b*) a side view of the same cell showing some of the actin cytoskeleton (green) and focal adhesions (brown) that form a bridge between the extracellular matrix (blue hatching) and the actin cytoskeleton; (*c*) the cell extends in a directed way by generating actin filaments that extend by the addition of actin monomers; (*d*) new focal adhesions form at the front and old ones dissolve at the rear of the cell; (*e*) a schematic of some of the modular proteins that make up a focal adhesions; A, actin; P, paxillin; T, talin; Fl, filamin; Fak, focal adhesion kinase; V, vinculin; PTP, phosphatases; Pk, PIP kinase; I*α* and I*β*, *α* and *β* subunits of integrin; Fn, part of fibronectin, a large protein in the extracellular matrix.

**Figure 4:**
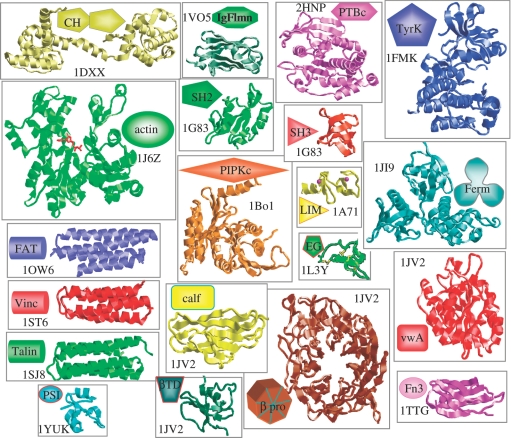
The structures of some of the modular protein units identified in figure 3*e*. The symbols generally correspond to those used in the SMART module database (http://smart.embl-heidelberg.de/). The structures were drawn using the graphics program Rastop (http:www.geneinfinity.org/rastop/); they can be seen to consist of a series of helices and strands. The LIM domain contains two zinc ions, coloured in magenta. PDB (http://www.rcsb.org/pdb/) accession codes, for the coordinates used to generate the protein representations, are shown for each structure. The structures were produced using Rastop (http://www.geneinfinity.org/rastop/manual/index.html).

**Figure 5:**
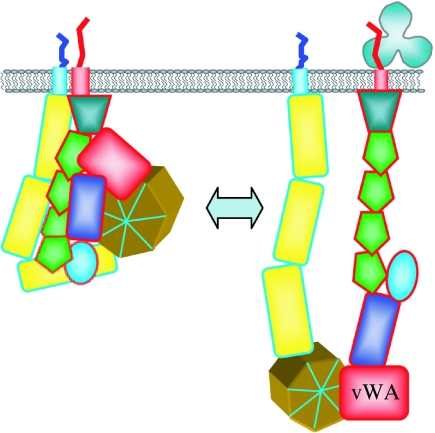
An illustration of two of the conformational states available to integrins. These correspond to an ‘off’ state on the left and an ‘on’ state, also shown in figure 3*e*. The large movements mainly arise from a rearrangement of the various modular units, but there is also a significant movement of a helical segment in the vWA domain in going from on state to the other (Luo & Springer 2006). The ‘on’ state can be stabilized by talin (cloverleaf structure) binding to the beta tail (red; Wegener *et al*. in press).

## Endnotes

1 ‘Progress in science depends on new techniques, new discoveries and new ideas, probably in that order’ attributed to Brenner (2002).

2 As an example of crude tools leading to crude models, Croone put forward a model of muscle contraction, based on the observation that bladders inflated by ‘several robust youths’ could exert a considerable force. He proposed that a chemical reaction took place between blood and nervous fluid that caused the muscle to swell and thus produce limb movement (Nayler & Maquet 2000). Models of muscle contraction have since improved (Geeves & Holmes 2005)!

3 In some ways the ability of NMR to solve structures is surprising since the wavelength of the applied radiation is much larger (more than 1 m) than the macromolecules studied (cf. microscopes where high resolution requires short wavelength). The required resolution actually arises from the local interactions between nuclear resonances that are assigned and resolved. In magnetic resonance imaging the resolution arises from the applied field gradients.
